# Integrated Network Pharmacology and Comprehensive Bioinformatics Identifying the Mechanisms and Molecular Targets of Yizhiqingxin Formula for Treatment of Comorbidity With Alzheimer’s Disease and Depression

**DOI:** 10.3389/fphar.2022.853375

**Published:** 2022-04-25

**Authors:** Tingting Zhang, Wei Wei, Surui Chang, Nanyang Liu, Hao Li

**Affiliations:** ^1^ Xiyuan Hospital, China Academy of Chinese Medical Sciences, Beijing, China; ^2^ College of First Clinical Medicine, Shandong University of Traditional Chinese Medicine, Jinan, China; ^3^ Wangjing Hospital, China Academy of Chinese Medical Sciences, Beijing, China

**Keywords:** Yizhiqingxin, Alzheimer’s disease, major depression disorder, network pharmacology, molecular docking

## Abstract

**Background:** The Yizhiqinxin formula (YZQX) has been used to treat Alzheimer’s disease (AD) or major depression disorder (MDD). However, its specific underlying mechanisms and therapeutic targets remain unclear.

**Methods:** The ingredients and putative targets of YZQX were screened using the TCMSP and Drugbank databases. Next, the GEO database was used to retrieve relevant differentially expressed genes (DEGs) in AD or MDD and normal tissues. The PPI network was established, merged, and further screened to identify the main ingredients and core targets of YZQX against AD and MDD comorbidities. We performed enrichment analysis of core targets to identify biological processes and pathways. Finally, AutoDock software was used to validate the binding affinity between the crucial targets of direct action and their corresponding ingredients.

**Results:** A total of 43 ingredients were identified from YZQX, of which 43 were screened to yield 504 targets. By establishing the PPI network, 92 targets were regarded as targets of YZQX against AD and MDD comorbidities in the core network. Promising targets (*HSP90AA1*, *ESR1*, *AKT1*, *VCAM1*, *EGFR*, *CDK1*, *MAPK1*, *CDK2*, *MYC*, *HSPB1*, and *HSPA5*) and signaling pathways (PI3K-Akt signaling pathway, ubiquitin-mediated proteolysis, MAPK signaling pathway, etc.) were filtered and refined to elucidate the underlying mechanism of YZQX against AD and MDD comorbidities. Molecular docking confirmed the ingredients of YZQX (quercetin and kaempferol) could bind well to multiple crucial targets.

**Conclusion:** The ingredients of YZQX, such as quercetin and kaempferol, might treat AD and MDD comorbidities by acting on multiple targets and pathways.

## Introduction

As a complex, multifactorial, progressive neurodegenerative disorder, Alzheimer’s disease (AD) is a major cause of dementia, resulting in the dysregulation of cognitive function, daily behavior, memory, and so on (Alexiou et al., 2019; Fiorini et al., 2020; Li et al., 2020). According to data from the Alzheimer’s Association Report, approximately 50 million people worldwide suffered from AD in 2018, and the number of AD patients is estimated to increase to 152 million by 2050 (2021). The gradually increasing morbidity of AD leads to an enormous economic and social burden, posing a significant challenge for both individuals and society (Uzun et al., 2011; Fransquet et al., 2018). Currently, therapeutic methods for AD included not only drug therapy but also non-pharmacological treatments of immunotherapy, natural products, and so on (Alexiou AT et al., 2019). It is well known that major depression disorder (MDD) is associated with an increased risk for onset of several neurological conditions, such as AD (Hayley, 2021). MDD is the most common psychiatric comorbidity in dementia, affecting approximately 50% of patients with AD (Chi et al., 2014; Romano et al., 2014). MDD is a heterogeneous disorder and accompanied by the primary symptoms of low mood, anhedonia, and loss of energy (Chi et al., 2014; Romano et al., 2014), as well as psychological signs and symptoms (American Psychiatric Association, 2013). An increasing body of evidence has shown that MDD could trigger, accompany, or aggravate the clinical hallmarks of dementia while accelerate cognitive decline, decreasing quality of life, and increasing mortality in patients with AD (Bennett and Thomas, 2014). Given these known associations and similar mechanisms of pathogenesis between depression and AD, it is necessary to reveal the biological mechanisms and develop effective treatment.

In recent years, increasing interest has been devoted to the use of traditional Chinese medicine (TCM) to prevent or treat chronic diseases because of their lower toxicity and more significant curative effects (Chao et al., 2017). YZQX formula, also known as Fuzheng Quxie Decoction, is a traditional Chinese herbal formula composed of three therapeutic herbs, a radix of Panax ginseng (Chinese name, Renshen), the rhizome of Coptis Chinensis (Chinese name, Huanglian), and Chuanxiong Rhizoma (Chinese name, Chuanxiong). Data from previous studies revealed that the YZQX formula could enhance learning and memory ability in SAMP8 mice and reduce tau hyperphosphorylation (Yang et al., 2017). This formula has also been shown to improve cognitive impairment and alleviate Aβ deposition by activating autophagy and the mTOR signaling pathway *in vivo* and *in vitro* (Yang et al., 2019). It is worth mentioning that ginseng treatment induces a variety of effects, including anti-depressive effects, in addition to suppressing or delaying the neurodegenerative process (Ong et al., 2015; Hou et al., 2020). In addition, Lee et al. studied the active ingredients of the YZQX formula, such as berberine, and found that they also exhibited antidepressant activities (Lee et al., 2012). However, at present, TCM plays a role mainly by acting on multiple ingredients, targets, and pathways. The specific mechanisms by which the YZQX formula prevents the co-occurrence of AD and MDD are still unclear.

**FIGURE 1:**
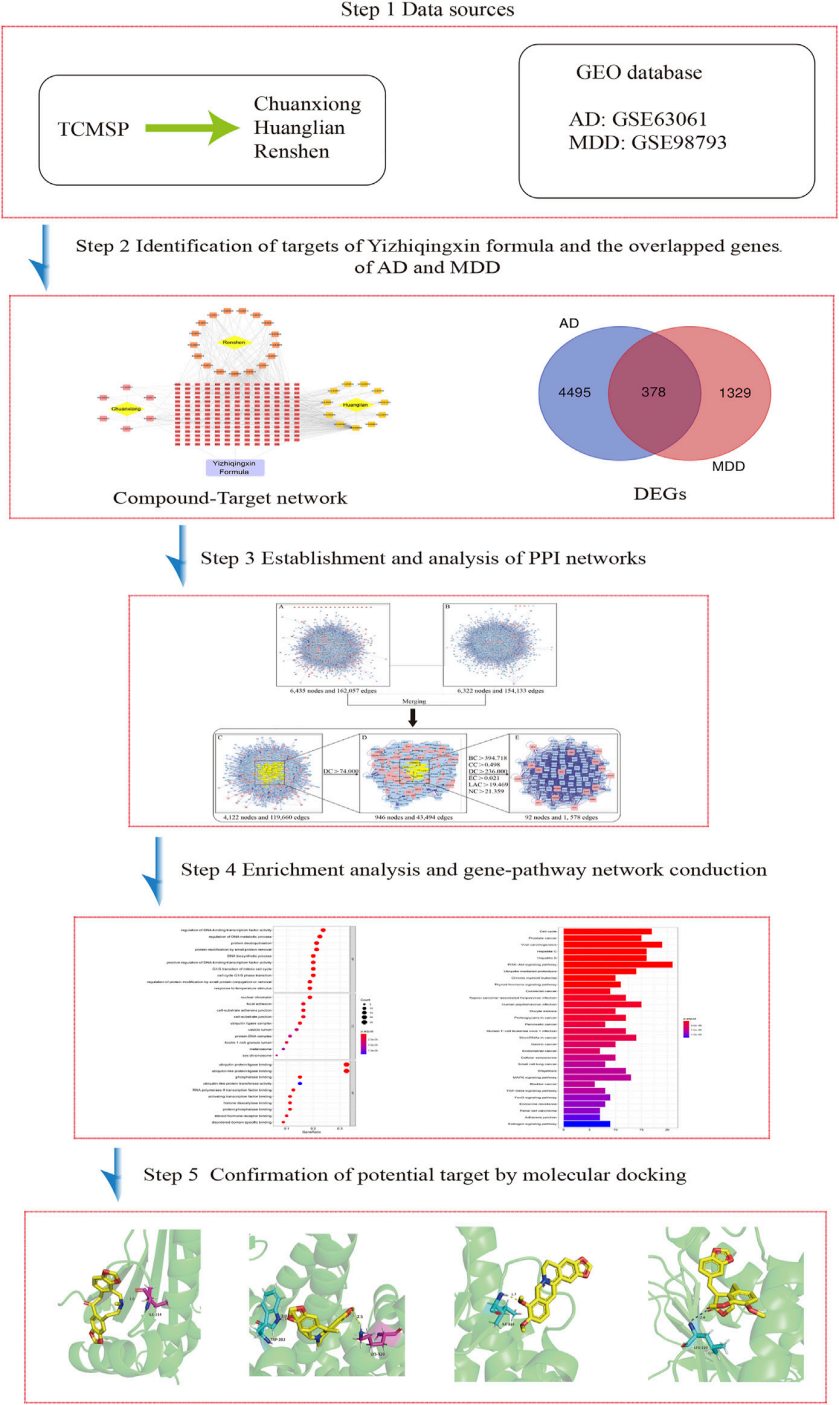
Workflow for Yizhiqingxin formula treatment of Alzheimer’s disease with depression.

Network pharmacology is a novel and powerful tool that could clarify the potential mechanisms of multiple ingredients, targets, and pathways of TCM (Berger and Iyengar, 2009). Molecular docking is a computer-aided drug design method that is frequently used to predict interactions and binding affinity between proteins and ligands (Kitchen et al., 2004). In recent years, molecular docking has grown into a popular method to explore and research TCM and their targets. The previous study has adopted molecular docking to explore the interaction between donepezil and human transferrin, which contributed to aid in having a better understanding of the activity and mechanism of protein and drug binding (Shamsi A et al., 2020). Therefore, in the present study, we adopted an integrated method of network pharmacology and molecular docking combined with high-throughput sequencing data to search for common targets and pathways for YZQX in the treatment of AD and MDD comorbidities and to mine the related biological mechanisms and signaling pathways. A flowchart of our study is presented in Figure 1.

## Methods

### Active Ingredients Identified by Yizhiqinxin Screening

Oral bioavailability (OB) and drug-likeness (DL) are the two main parameters of the absorption, distribution, metabolism, and excretion (ADME) model (Barton et al., 2006) that affect drug absorption throughout the gastrointestinal tract. Hence, by using “Renshen,” “Huanglian,” and “Chuanxiong,” as keywords, we identified the active ingredients of the YZQX formula, screening for compounds with oral bioavailability (OB) ≥30% and drug-likeness (DL) ≥0.18 in the Traditional Chinese Medicine Systems Pharmacology (https://tcmspw.com/tcmsp.php) (Ru et al., 2014).

### Collection of Putative Targets

After screening the active ingredients of the YZQX formula, the next significant process was to identify their corresponding targets. The putative targets of the active ingredients in the YZQX formula were identified and collected using the DrugBank database (https://www.drugbank.ca/) (Law et al., 2014). Further, all of these obtained targets were limited to the species “*Homo sapiens*” in the Uniprot database (https://www.uniprot.org/) (2017). In addition, we used the Cytoscape 3.7.2 software to construct and present a Herb-ingredient-target network of the YZQX formula. In this network, nodes represent formulas, herbs, ingredients, and targets, while the edges represent their interaction relationships.

### Search, Identification, and Analysis of Differentially Expressed Genes in Alzheimer’s Disease and Major Depression Disorder

The microarray data of GSE63061 and GSE98793 were downloaded from the GEO database (http://www.ncbi.nlm.nih.gov/geo/) using their microarray platforms, GPL10558 (Illumina HumanHT-12 V4.0 expression bead chip) and GPL570 (Affymetrix Human Gene Expression Array), respectively. Among them, the GSE63061 dataset contained 139 AD samples and 134 normal samples, and the GSE98793 dataset contained 128 MDD samples and 64 normal samples. Thereafter, the probes were converted into gene symbols according to the annotation information of the corresponding platform. The DEGs between disease (AD or MDD) groups and normal groups were identified and screened as implemented in the R software (Version 3.6.0) limma package with a threshold value of *p* < 0.05. DEGs with *p* < 0.05, and |log2 fold change (FC)| > 0.1 were then presented visually using volcano plots. Venn diagrams were used to further obtain the overlapping genes from the AD and MDD microarray datasets.

### Construction of the Protein-Protein Interaction Network

To explore all the possible interactions among genes, the PPI network of YZQX-related targets and AD-MDD overlapped targets were established using the Cytoscape plugin BisoGenet (Martin et al., 2010), which integrates PPI data from the following six databases: the Biological General Repository for Interaction Datasets (BioGRID), the Biomolecular Interaction Network Database (BIND), the Database of Interacting Proteins (DIP), the Molecular INTeraction Database (MINT), the Human Protein Reference Database (HPRD), and the IntAct Molecular Interaction Database (IntAct).

### Merging and Analysis of Protein-Protein Interaction Networks

Building on the previous process, two PPI networks were intersected into a merged network for further analysis using Cytoscape 3.7.2 software. Next, the CytoNCA (Tang et al., 2015) plugin of Cytoscape was used to calculate and evaluate the topological significance of each node in the network based on these parameters of degree centrality (DC), betweenness centrality (BC), closeness centrality (CC), eigenvector centrality (EC), local average connectivity-based method (LAC), and network centrality (NC). A previous study (Tang et al., 2015) specified in detail the definitions and computing methods of these parameters; in brief, a higher value represents greater importance of the node in the merged network. We calculated the degree of centrality in a merged network. It is worth mentioning that this gene is considered “a big hub” when the degree centrality of one node is more than twice the median degree centrality in a merged network. Subsequently, a sub-network was extracted based on two times the median degree centrality and used to perform the next screening. The median values of the other five parameters served as threshold values to further identify the hub genes and core network. Finally, after two screenings, the core network of YZQX treating comorbidity with AD and MDD was obtained.

In addition, we also further analyzed the attributes of network screened by CytoNCA using MCODE plugins in Cytoscape software.

### Enrichment Analysis of the Core Network

ClusterProfiler (Yu et al., 2012), a new ontology-based package of R software version 3.6.0, which assigned the GO and Kyoto Encyclopaedia of Genes and Genomes (KEGG) database, was used for enrichment analysis. Of note, the parameter was set to a *p*-value cutoff <0.05, which was deemed to be significant. The GO term contained three categories: biological process (BP), cellular component (CC), and molecular function (MF). The results of GO, sorted from small to large according to the *p*-value as a parameter, and the top 10 terms of each category were selected using a bubble plot for visualization. For the top five terms, a sub-network was then established in Cytoscape software version 3.7.2 to visualize the relationships between genes and GO terms. Furthermore, based on the genes of the core network of YZQX in AD and MDD treatment, KEGG pathway analysis was performed, and a bubble plot was plotted.

### Molecular Docking Verification

To explore the possible docking modes and binding affinities between macromolecules and small molecules (ligands) in-depth, we performed molecular docking based on molecular modeling techniques using AutoDock software (Yang et al., 2016). We subsequently identified the direct-acting targets in the core network and their corresponding bioactive ingredients for molecular docking. The X-ray crystal structures of key targets were obtained from the RCSB Protein Data Bank (PDB, https://www.rcsb.org/) and saved in PDB format. The MOL2 formats of the bioactive ingredients were downloaded from the ZINC database (http://zinc.docking.org/). Before molecular docking, we used AutoDock software (version 4.2.6) to remove the ligand, hydrogenate, calculate the charge, add protein type for the macromolecules, and saved it in PDBQT format. Subsequently, we ran the AutoDock to perform molecular docking between macromolecules and ligands based on the default parameters. Blinding energy of less than “−5” represented a better binding interaction between macromolecules and small molecules (Gaillard, 2018). Finally, the PyMOL software (version 2.4.1) was used to visualize the docking results.

## Results

### Ingredients and Their Putative Targets of Yizhiqinxin and Ingredient-Target Network

A total of 43 ingredients in YZQX were screened from the TCMSP database with the criteria of OB ≥ 30% and DL ≥ 0.18. These were defined as candidate bioactive ingredients, of which seven were from Chuanxiong, 14 from Huanglian, and 22 from Renshen. The detailed characteristics, including molecular ID, molecular name, structure, OB, and DL, of the selected bioactive ingredients are presented in Table 1. We retrieved the DrugBank database using candidate bioactive ingredients and identified 504 ingredient-related targets. From each of the Chinese herbs in YZQX, 39, 251, and 214 potential targets were obtained, respectively (Supplementary Table S1). After removing the reduplicated targets, the total number of putative targets associated with bioactive ingredients was adjusted to 193. In addition, these targets were annotated as gene symbols in the UniProt database.

**TABLE 1:** The final selected ingredients in YZQX for analysis.

Drug	Molecular ID	Molecular name	CAS	Structure	OB (%)	DL
Chuanxiong	MOL001494	Mandenol	544-35-4	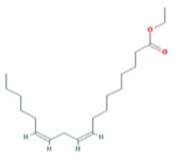	42.00	0.19
	MOL002135	Myricanone	32492-74-3	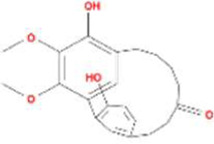	40.60	0.51
	MOL002140	Perlolyrine	29700-20-7	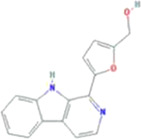	65.95	0.27
	MOL002151	senkyunone	142182-61-4	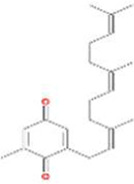	47.66	0.24
	MOL002157	wallichilide	93236-64-7	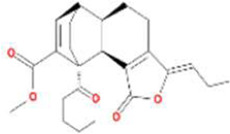	42.31	0.71
	MOL000359	sitosterol	83-46-5	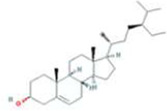	36.91	0.75
	MOL000433	FA	33609-88-0	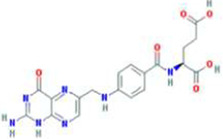	68.96	0.71
Huanglian	MOL001454	berberine	633-66-9	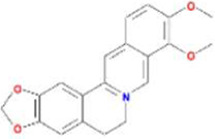	36.86	0.78
	MOL013352	Obacunone	751-03-1	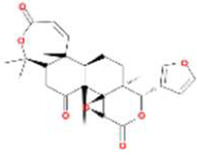	43.29	0.77
	MOL002894	berberrubine	1540-69-1	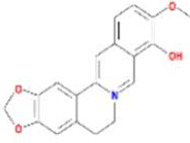	35.74	0.73
	MOL002897	epiberberine	1816598	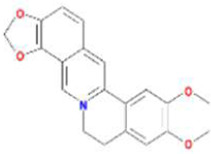	43.09	0.78
	MOL002903	(R)-Canadine	522-97-4	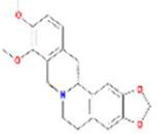	55.37	0.77
	MOL002904	Berlambine	549-21-3	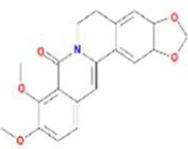	36.68	0.82
	MOL002907	Corchoroside A_qt	NA	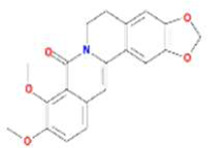	104.95	0.78
	MOL000622	Magnograndiolide	92618-98-9	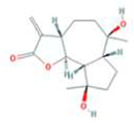	63.71	0.19
	MOL000762	Palmidin A	17062-55-4	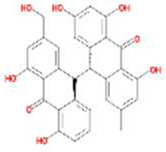	35.36	0.65
	MOL000785	palmatine	3486-67-7	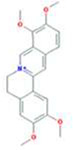	64.60	0.65
	MOL000098	quercetin	73123-10-1	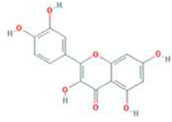	46.43	0.28
	MOL001458	coptisine	3486-66-6	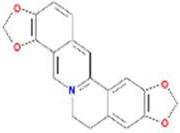	30.67	0.86
	MOL002668	Worenine	38763-29-0	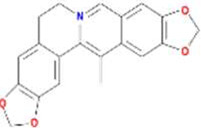	45.83	0.87
	MOL008647	Moupinamide	66648-43-9	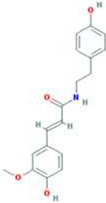	86.71	0.26
Renshen	MOL002879	Diop	25103-50-8	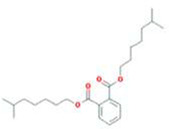	43.59	0.39
	MOL000449	Stigmasterol	83-48-7	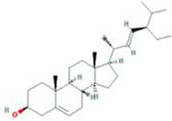	43.83	0.76
	MOL000358	beta-sitosterol	83-46-5	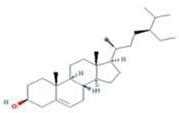	36.91	0.75
	MOL003648	Inermin	19908-48-6	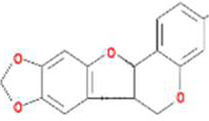	65.83	0.54
	MOL000422	kaempferol	520-18-3	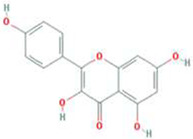	41.88	0.24
	MOL004492	Chrysanthemaxanthin	26989-20-8	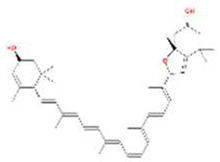	38.72	0.58
	MOL005308	Aposiopolamine	NA	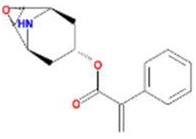	66.65	0.22
	MOL005314	Celabenzine	53938-08-2	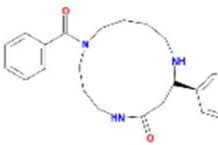	101.88	0.49
	MOL005317	Deoxyharringtonine	36804-95-2	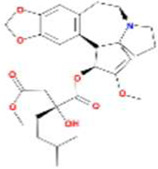	39.27	0.81
	MOL005318	Dianthramine	136945-65-8	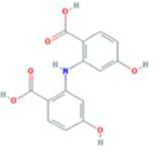	40.45	0.20
	MOL005320	arachidonate	506-32-1	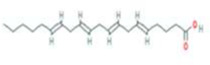	45.57	0.20
	MOL005321	Frutinone A	38210-27-4	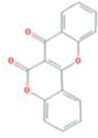	65.90	0.34
	MOL005344	ginsenoside rh2	78214-33-2	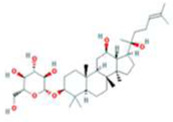	36.32	0.56
	MOL005348	Ginsenoside-Rh4_qt	174721-08-5	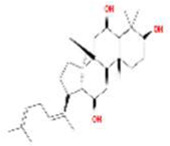	31.11	0.78
	MOL005356	Girinimbin	23095-44-5	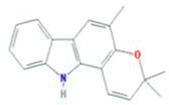	61.22	0.31
	MOL005357	Gomisin B	58546-55-7	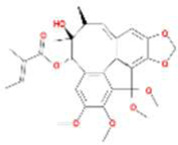	31.99	0.83
	MOL005360	malkangunin	52691-06-2	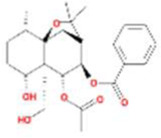	57.71	0.63
	MOL005376	Panaxadiol	19666-76-3	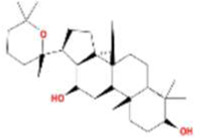	33.09	0.79
	MOL005384	suchilactone	50816-74-5	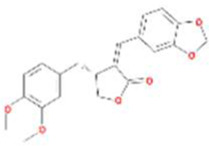	57.52	0.56
	MOL005399	alexandrin_qt	474-58-8	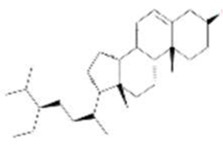	36.91	0.75
	MOL005401	ginsenoside Rg5_qt	186763-78-0	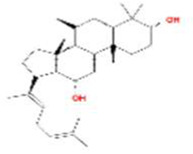	39.56	0.79
	MOL000787	Fumarine	130-86-9	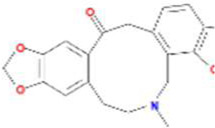	59.26	0.83

OB, oral bioavailability; DL, drug-like.

### Construction of Ingredient-Target Network in Yizhiqinxin

**FIGURE 2:**
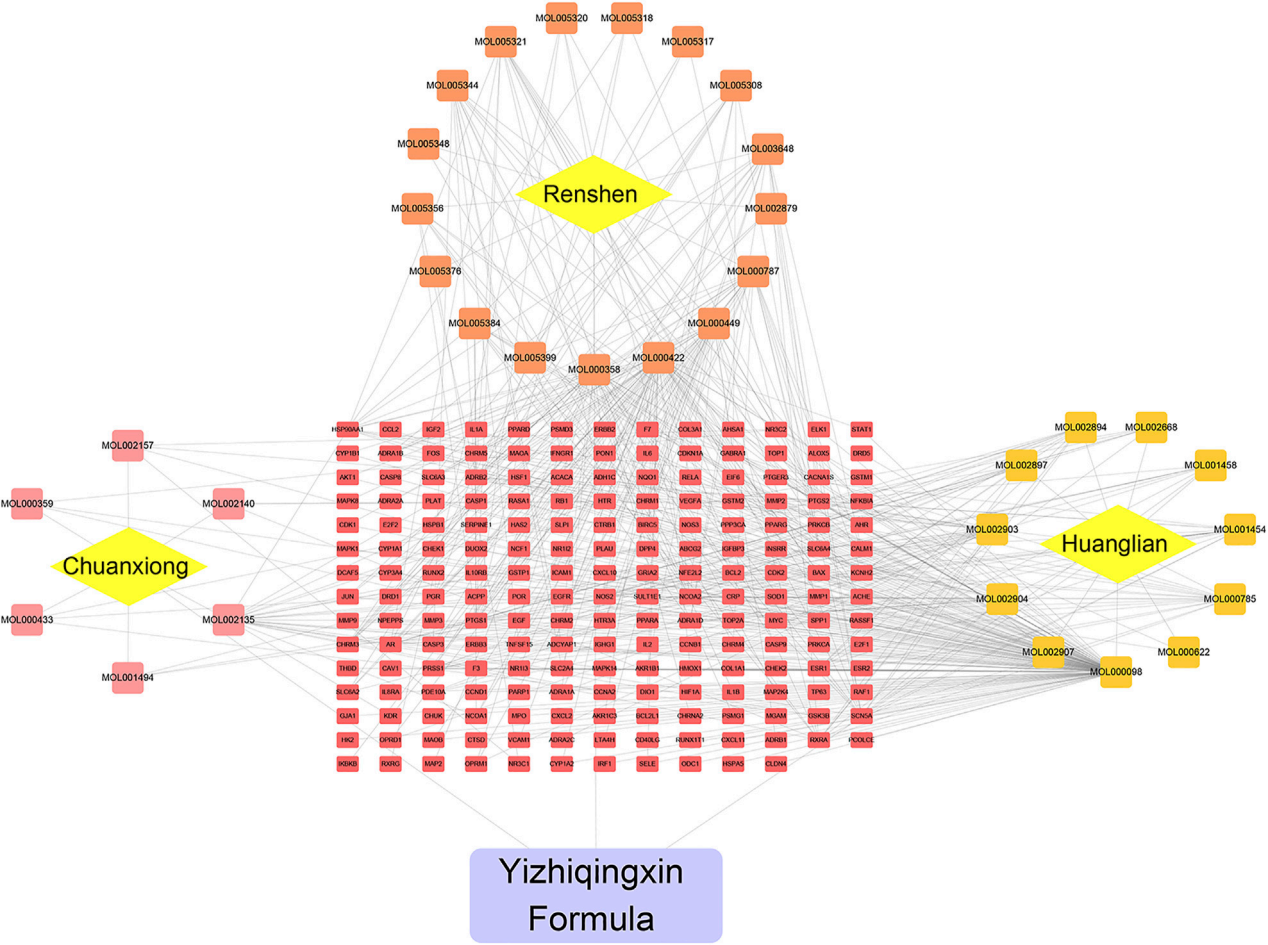
Herb-Ingredient-target network of YZQX. Yellow rhombus represents herb, the square represents ingredients, the red rectangle represents targets, the purple rectangle represents the YZQX formula.

Subsequently, using Cytoscape software, we established a network indicating the relationship among Chinese herbs, ingredients, and putative targets, as shown in Figure 2. This ingredient-target network contained 231 nodes and 541 edges. Quercetin was the most prolific, with 141 potential targets, followed by kaempferol (56), and beta-sitosterol (28).

### Identification of Alzheimer’s Disease-Related and Major Depression Disorder-Related Differentially Expressed Genes

**FIGURE 3:**
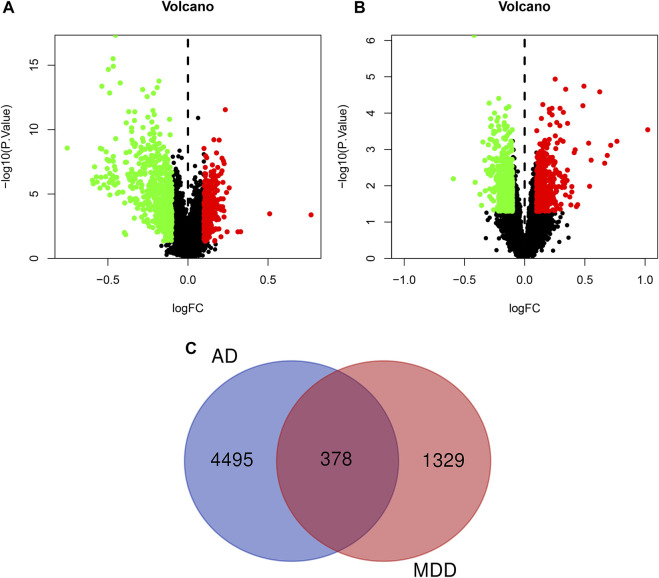
Volcano plot of differentially expressed genes in AD **(A)** and depression **(B)**. The red dots represent significantly up-regulated genes, the green dots represent significantly down-regulated genes. Panel **(C)** refers to the overlapped genes of AD and depression.

We downloaded the AD expression datasets and selected DEGs by calculating the difference in gene expression between 134 control samples and 139 AD samples. Ultimately, 4,873 DEGs, including 2,104 downregulated and 2,769 upregulated genes, were identified from the GSE63061 dataset using the limma package in R software. Additionally, based on the cutoff criterion of *p <* 0.05, a total of 1,707 DEGs, containing 889 downregulated and 818 upregulated genes, were extracted and analyzed from the microarray data GSE98793. The DEGs with *p <* 0.05, and |log2 FC| > 0.1 in AD or MDD were visualized in a volcano plot (Figures 3A,B). And the detailed information of AD- and MDD-related DEGs was presented in Supplementary Table S2. Moreover, the overlapping genes of AD and MDD were 378, as shown in Figure 3C.

### Merging and Analysis of Protein-Protein Interaction Networks

**FIGURE 4:**
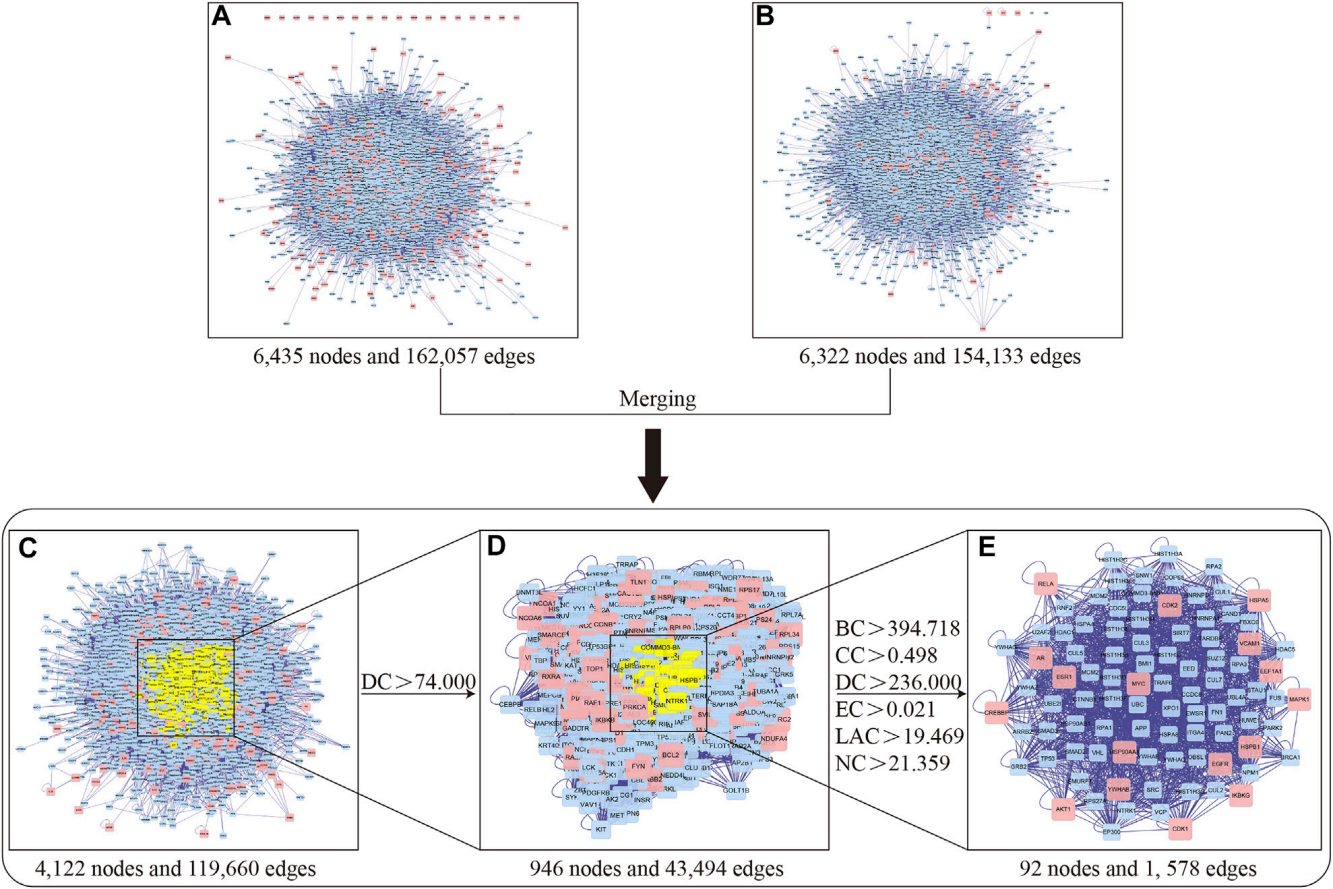
Identification of core network and targets of YZQX against AD with depression. **(A)** AD with depression-related targets PPI network. **(B)** YZQX putative targets PPI network. **(C)** The interactive PPI network of YZQX putative targets and AD with depression-related targets. **(D)** PPI network of significant targets extracted from Panel **(C)**. **(E)** PPI network of candidate YZQX targets for AD with depression treatment extracted from Panel **(D)**. Notes: AD, Alzheimer’s disease; DC, degree centrality; BC, betweenness centrality; CC, closeness centrality; EC, eigenvector centrality; LAC, local average connectivity-based method; NC, network centrality.

In the present study, we used the BisoGenet plugin based on the six PPI databases to generate PPI networks of YZQX putative targets and comorbidity with AD- and MDD-related targets (Figure 4). As presented in Figure 4A, the PPI network of AD with depression-related targets consisted of 162,057 edges and 6,435 nodes. The PPI network of putative targets associated with YZQX contained 154,133 edges and 6,322 nodes (Figure 4B).

To explore the pharmacological mechanisms of YZQX in treating AD and MDD comorbidities, we utilized the merge network function supplied by Cytoscape software to generate a new merged network, with 119,660 edges and 4,122 nodes, consisting of overlapping genes from the two networks mentioned above (Figure 4C). After DC calculation, 946 nodes and 43,494 edges with a DC of more than two-fold the median were selected for integration into the subnetwork (Figure 4D). Next, these genes were further screened based on topological property analysis of DC, BC, CC, EC, LAC, and NC using the CytoNCA plugin in the Cytoscape software. The median values showed BC values of 394.718, CC of 0.498, EC of 0.021, NC of 21.359, LAC of 19.469, and the two-fold median DC of 236 (Figure 4E), and finally, a core network including 92 targets together with 1,578 edges was identified for YZQX against AD and MDD comorbidities. The detailed topology parameter of the core network involving 92 targets is presented in the Supplementary Table S3.

According to the ranking of MCODE score, the largest cluster has 25 nodes and 215 edges, with a score of 17.917 points; The smallest module has three nodes and three edges, with a score of three points. And the clusters are presented in Supplementary Figure S1.

### Enrichment Analysis of Core Targets

**FIGURE 5:**
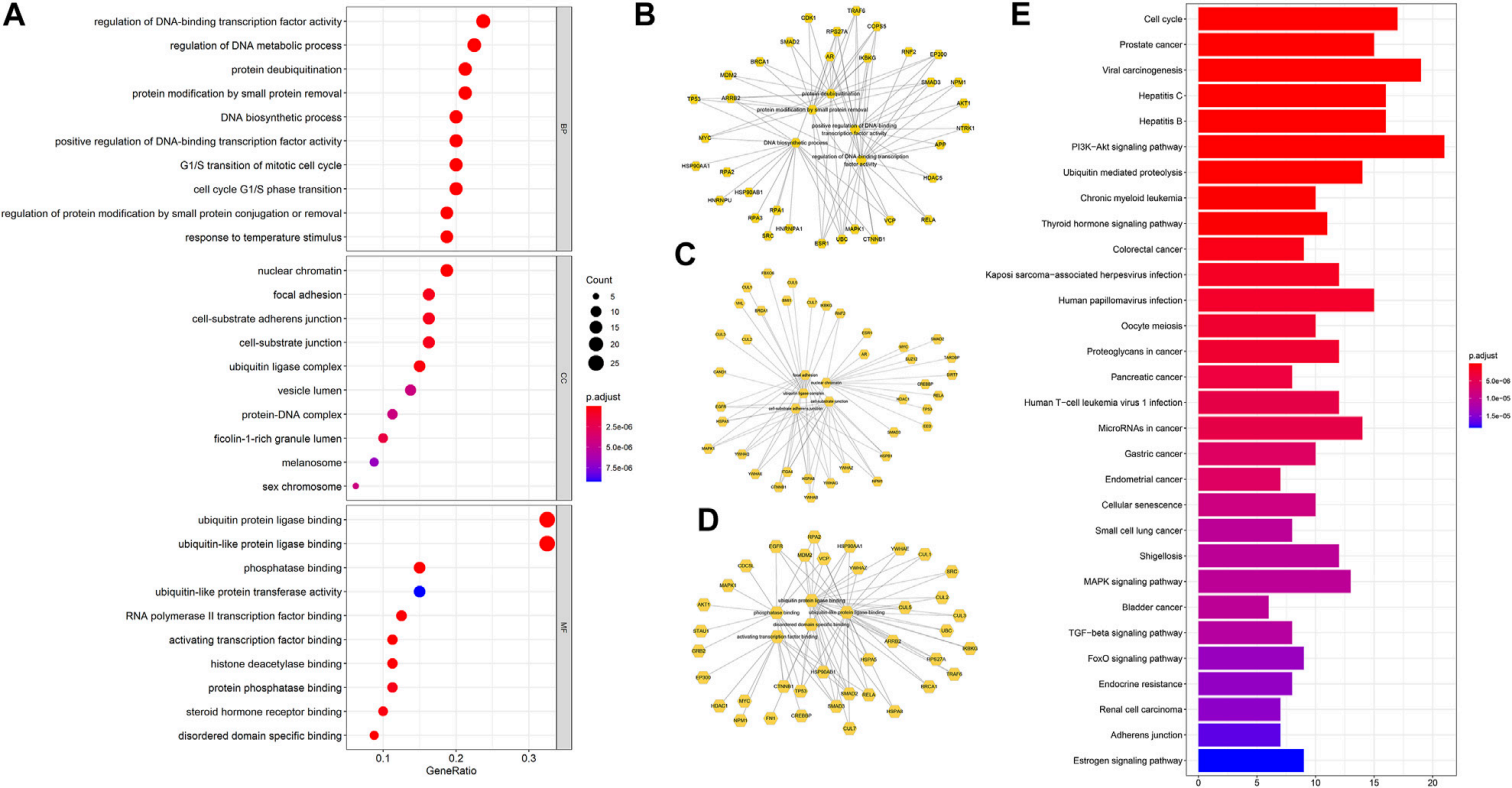
The top 10 terms of GO analysis about targets involved in the core network **(A)**. **(B)** Sub-network showing the top five BP terms and related genes; **(C)** Sub-network showing the top 5 CC terms and related genes; **(D)** Sub-network showing the top five MF terms and related genes. **(E)**The top 30 KEGG pathway enrichment of core targets of YZQX against AD with depression. Pathways that had significant changes of p. adjust <0.05 were identified. The dot size represents a number of genes and color represents the p. adjust value.

To reveal the biological mechanisms of the 92 crucial targets, the cluster profile package in R software was used to perform GO and KEGG enrichment analysis, yielding a total of 1,212 BPs, 95 CCs, 119 MFs, and 117 KEGG pathways (*p* < 0.05) (Supplementary Tables S4, S5). As indicated in Figure 5, the highly enriched GO terms in BP were involved in DNA-binding transcription factor activity regulation, DNA metabolic process regulation, protein deubiquitination, protein modification by small protein removal, and the DNA biosynthetic process. The main CCs of the 92 core targets were related to nuclear chromatin, focal adhesion, cell-substrate adherens junction, cell-substrate junction, and ubiquitin ligase complex. MFs focused on ubiquitin-protein ligase binding, ubiquitin-like protein ligase binding, phosphatase binding, ubiquitin-like protein transferase activity, and RNA polymerase II transcription factor binding. In addition, based on the KEGG pathway data analysis, we found that the PI3K-Akt signaling pathway, ubiquitin-mediated proteolysis, estrogen signaling pathway, MAPK signaling pathway, and TGF-beta signaling pathway concurrently occupied the process of YZQX treatment of comorbidity with AD and MDD (Figure 5E).

### Validation of Molecular Docking

**FIGURE 6:**
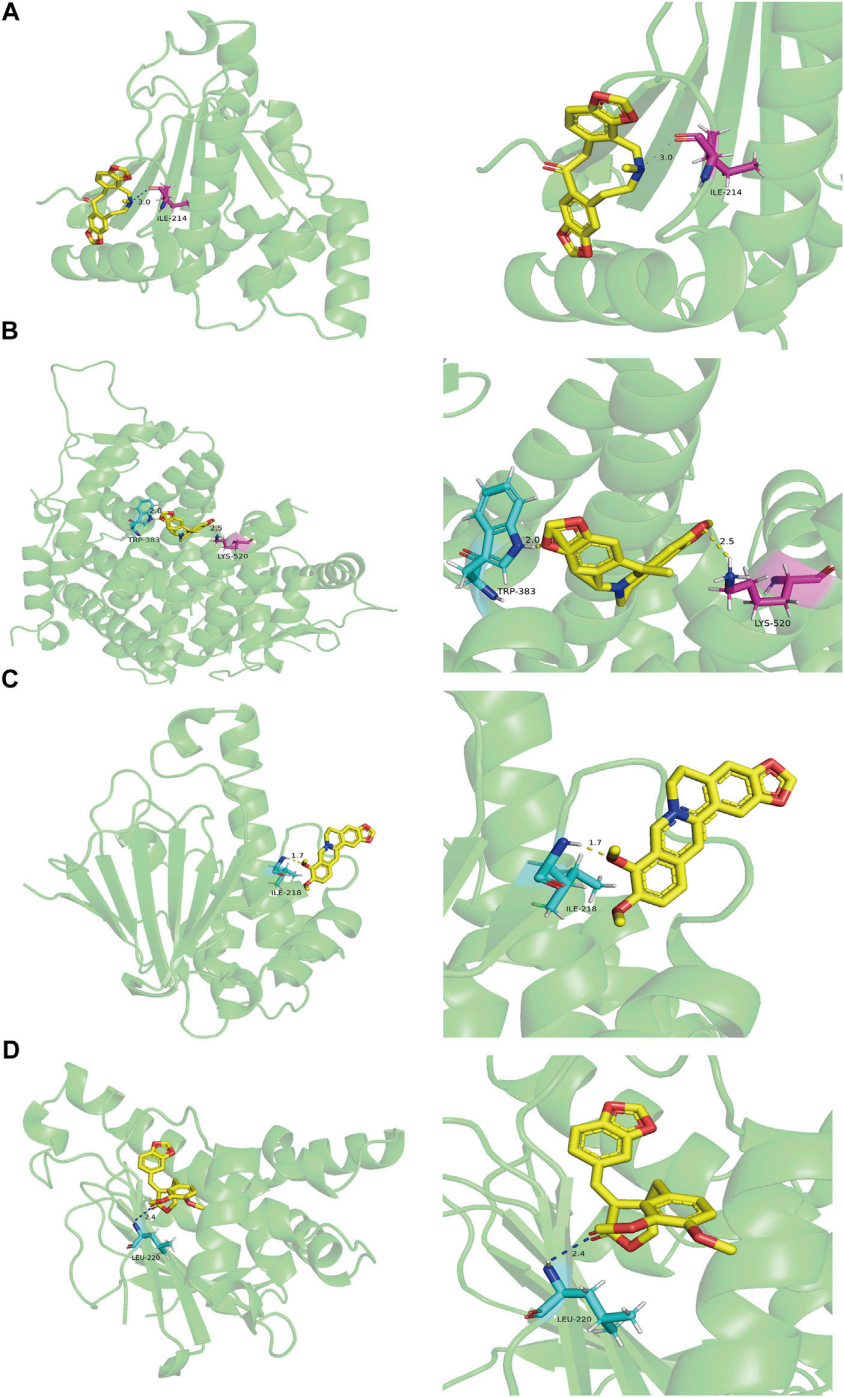
The molecular docking results of key targets and their corresponding ingredients. **(A)** Fumarine and HSP90AA1 **(B)** Worenine and ESR1 **(C)** berberine and HSP90AA1 **(D)** suchilactone and HSP90AA1.

The PPI network indicated that *HSP90AA1*, *ESR1*, *AKT1*, *VCAM1*, *EGFR*, *CDK1*, *MAPK1*, *CDK2*, *MYC*, *HSPB1*, and *HSPA5* are pivotal targets in the core network mentioned above, and these nodes were also overlapping genes of AD and MDD comorbidity. In accordance with key targets, we worked backward to their corresponding bioactive ingredients serving as ligands, thus performing validation of molecular docking. In total, 2 ingredients were docked with *AKT1*, 2 with *CDK1*, 3 with *CDK2*, 6 with *ESR1*, 11 with *HSP90AA1*, 1 with *EGFR*, 1 with *HSPA5*, 1 with *HSPB1*, 1 with *MAPK1*, 1 with *MYC*, and 1 with *VCAM1*. The ligand-receptor binding conformation had the lowest binding energy, indicating that this conformation is the most stable, and thus the possibility of interaction is the greatest. The top three docking combinations for the target macromolecules and corresponding ingredients included Fumarine and *HSP90AA1*, Worenine and *ESR1*, berberine and *HSP90AA1*, with binding energies of −6.83, −6.6, and −6.26 kcal/mol, respectively. The other specific docking scores of the ingredients with targets are shown in Supplementary Table S6. If the docking score was less than −6 kcal/mol and ranked as the top 4, PyMOL was used to display the three-dimensional view of the docking mode of the ingredients and the target (Figure 6).

## Discussion

Depression is recognized as the most common comorbidity of AD and affects the treatment of AD patients (Chi et al., 2014; Romano et al., 2014). Hence, the development of an effective treatment for patients with AD and MDD comorbidity is extremely important. As a traditional Chinese medicine, the formula or its main ingredients in YZQX have been widely used to treat comorbid AD and MDD with remarkable clinical effects (Lee et al., 2012; Ong et al., 2015; Yang et al., 2017; Yang et al., 2019; Hou et al., 2020). In this present study, we coupled network pharmacology with GEO database analysis to explore the common mechanisms of YZQX in treating AD and MDD comorbidity. By establishing and screening a PPI network of the overlapping targets of YZQX and AD as well as MDD, 92 targets were identified and considered as core genes. Of these, *HSP90AA1*, *ESR1*, *AKT1*, *VCAM1*, *EGFR*, *CDK1*, *MAPK1*, *CDK2*, *MYC*, *HSPB1*, and *HSPA5* acted directly on these diseases. Further, KEGG pathway analysis revealed that these core targets were mainly related to the PI3K-Akt signalling pathway, ubiquitin-mediated proteolysis, estrogen signalling pathway, MAPK signalling pathway, TGF-beta signalling pathway, etc. The abovementioned evidence suggests the clinically promising potential of YZQX in the treatment of AD comorbidity with depression.

In this study, we identified 43 ingredients in YZQX, and molecular docking was performed to compare these crucial targets with the corresponding ingredients. Quercetin and kaempferol could bind well to multiple crucial targets, which served as the main bioactive ingredients. Quercetin is a predominant flavonoid that has multiple beneficial properties, including antioxidative and anti-inflammatory effects (Brüll et al., 2015). Moreover, data from previous studies revealed its neuroprotective effects on AD (Rishitha and Muthuraman, 2018), which may be ascribed to its ability to cross the blood-brain barrier (BBB) and exert antioxidant and anti-inflammatory effects in the brain (Youdim et al., 2004). Likewise, quercetin also markedly promoted neurogenesis by increasing the level of doublecortin and subsequently alleviating depressive-like behavior (Mehta et al., 2017). Additionally, in previous network pharmacology studies, quercetin was also found to be an ingredient that contributed significantly in the treatment of comorbidity with AD and MDD, participating in polypharmacological and synergistic mechanisms (Qi et al., 2020; Yuan et al., 2020). Kaempferol is a natural flavonoid, which is found in numerous fruits and vegetables and can trigger multiple biological mechanisms, including anti-inflammation, antioxidant activity, neuroprotective effect, and antidepressant effects (Kim et al., 2007; Kim et al., 2008; Silva Dos Santos et al., 2020). An *in vitro* study on rats indicated that kaempferol improved the cognitive function in AD rat models, thereby exerting its neuroprotective effect (Zarei et al., 2019). Likewise, Gao et al. have shown that kaempferol also plays a role in antidepressant capacities mainly mediated through its anti-inflammatory and antioxidant effect which work by enhancing the AKT/β-catenin signaling pathway (Gao et al., 2019). Of note, substantive evidence is accumulating to suggest that treatments with anti-inflammatory and anti-depressant agents may also reduce or prevent dementia in patients with MDD (Hayley, 2021).

Moreover, in this study, PPI networks between the ingredient-target of YZQX and comorbidity with AD and MDD were established using Cytoscape, followed by merging and topological parameters. Eventually, 92 targets were identified and enrichment analysis was performed to further clarify the underlying mechanisms of YZQX treatment in patients with comorbid AD and MDD. KEGG analysis indicated that 92 targets were mainly related to the following pathways: PI3K-Akt signalling pathway, ubiquitin-mediated proteolysis, estrogen signalling pathway, MAPK signalling pathway, TGF-beta signalling pathway, etc. Converging reports on the PI3K-Akt signaling pathway have shown that it plays a crucial role in multiple processes of cell reproduction, differentiation, and apoptosis. Further enhancing PI3K/Akt function may prevent the development of AD by alleviating the downstream effects of Aβ (Curtis and Bandyopadhyay, 2021). Moreover, when the PI3K/Akt signaling axis in neurons is activated, it could reduce Tau hyperphosphorylation by suppressing GSK-3β activity (Koh et al., 2008; O’Neill et al., 2012; O’Neill, 2013). More importantly, this was also associated with neuroplasticity and could exert an anti-depressive effect by strengthening synapse formation and axon dendrite extension (Singh et al., 2017; Ali et al., 2018). Relevant research has also found that several neurotrophins, particularly brain-derived neurotrophic factor (BDNF), play a key role in the pathogenesis of MDD (Wiener et al., 2015; Castrén and Kojima, 2017). Additionally, BDNF activates the PI3K/Akt pathway, thereby increasing the length and complexity of dendrites and regulating neuronal survival (Jaworski et al., 2005; Kumar et al., 2005). Growing evidence now indicates that the MAPK signaling pathway plays a crucial role in a variety of biological processes and is capable of regulating inflammation and the stress response (Venigalla and Turner, 2012). It is worth mentioning that this pathway is closely associated with not only neuron apoptosis, but also β-amyloid deposition and tau hyperphosphorylation in AD (Song et al., 2014; Kheiri et al., 2018). In addition, the differential expression of miRNA profiles in rat models of depression also concentrates on the MAPK pathway and thereby affects the pathogenesis of depression (Fang et al., 2020).

Some hub targets predicted by molecular docking in this study have also been reported in previous studies (Pirskanen et al., 2005; Sulistio and Heese, 2016; Lackie et al., 2017). ESR1, located on chromosome 6q25, is an essential mediator of the hormonal response in estrogen-sensitive tissues. During the course of AD, estrogen could help in the following activities: promoting neuronal cell survival, alleviating neuronal damage, protecting the neuronal cell from neurotoxins, and strengthening synaptic transmission and neurogenesis (Pirskanen et al., 2005). When binding with estrogen, *ESR1* can regulate gene expression and its corresponding function *via* the interactions between regulatory regions and target genes (Xu et al., 2013). A study of the relevant literature revealed that *ESR1* serves as a key factor of depression, particularly in women (Ancelin et al., 2007; Osterlund, 2010; Ryan et al., 2012). Interestingly, *ESR1* can control the activity of serotonin-activated neurons by regulating the quantity and function of serotonin receptors and controlling neurotransmitters (Summer and Fink, 1995; Andrade et al., 2005). *HSP90AA1*, a member of the heat shock family, is highly conserved in eukaryotes. Of particular interest was the finding that HSPs are also involved in regulating the stabilization and function of p-tau in AD (Sulistio and Heese, 2016; Lackie et al., 2017). Apart from this, they also play a key role in protein folding and proteosomal degradation (Lackie et al., 2017).

In the present study, the underlying mechanisms and molecular targets of YZQX for AD with depression were illustrated using integrated network pharmacology and molecular docking approach. YZQX may exert its effects *via* action on the PI3K-Akt signalling pathway, ubiquitin-mediated proteolysis, Estrogen signalling pathway, MAPK signalling pathway, and TGF-beta signalling pathway. This study further identified *HSP90AA1*, *ESR1*, *AKT1*, *VCAM1*, *EGFR*, *CDK1*, *MAPK1*, *CDK2*, *MYC*, *HSPB1*, and *HSPA5* as the key targets of YZQX in the treatment of AD with comorbid depression. This study further confirmed that YZQX is a promising therapeutic method for the development of safe and effective multitarget for AD with depression.

## Data Availability

The original contributions presented in the study are included in the article/Supplementary Material, further inquiries can be directed to the corresponding authors.
