# Supplementary figures and images for: Integrated Network Pharmacology and Comprehensive Bioinformatics Identifying the Mechanisms and Molecular Targets of Yizhiqingxin Formula for Treatment of Comorbidity With Alzheimer’s Disease and Depression
Source: Front Pharmacol. 2022 Apr 25;13:853375. doi: 10.3389/fphar.2022.853375 (PMC9081443; doi:10.3389/fphar.2022.853375)

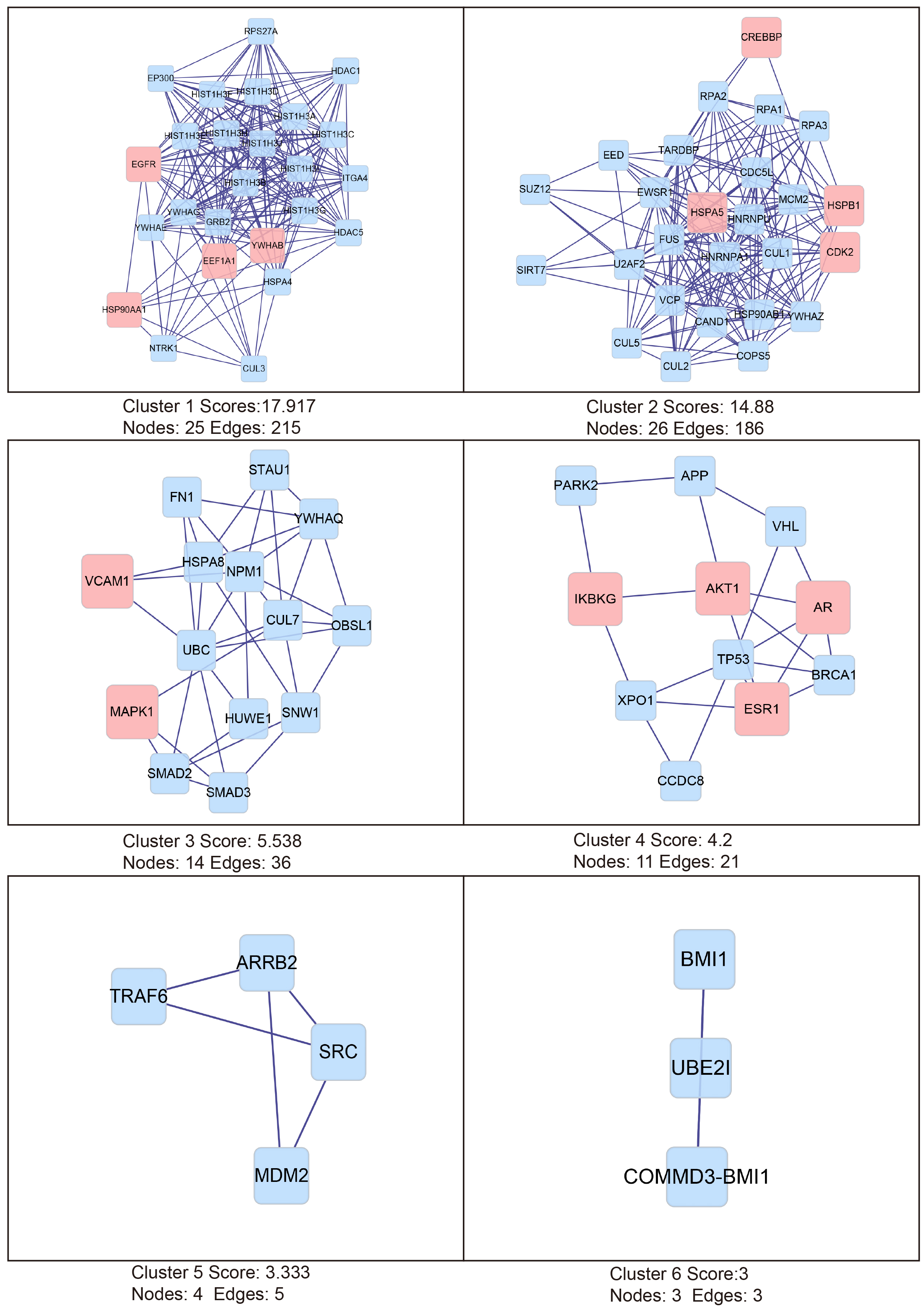

Supplement: Supplementary file 2 [file Image1.JPEG]
