# Supplementary material for: Integrated Network Pharmacology and Comprehensive Bioinformatics Identifying the Mechanisms and Molecular Targets of Yizhiqingxin Formula for Treatment of Comorbidity With Alzheimer’s Disease and Depression
Source: Front Pharmacol. 2022 Apr 25;13:853375. doi: 10.3389/fphar.2022.853375 (PMC9081443; doi:10.3389/fphar.2022.853375)
Supplement: Supplementary file 5 [file Table3.DOCX]

| The topological parameters of 92 targets in core network | | | | | | |
| --- | --- | --- | --- | --- | --- | --- |
| Gene | BC | CC | DC | EC | LAC | NC |
| MAPK1 | 1468.522727 | 0.533898 | 326 | 0.027742 | 25.35772 | 93.44753086 |
| YWHAG | 2139.30538 | 0.545612 | 316 | 0.04562 | 34.45283 | 126.2834395 |
| YWHAE | 1845.854952 | 0.546243 | 302 | 0.050529 | 37.91925 | 136.62 |
| AR | 2535.362812 | 0.54248 | 286 | 0.030928 | 27.61745 | 114.7394366 |
| EGFR | 7733.349486 | 0.573422 | 675 | 0.052708 | 34.107 | 86.08469539 |
| YWHAB | 1423.222817 | 0.533597 | 293 | 0.031939 | 26.39669 | 109.604811 |
| ITGA4 | 6706.497111 | 0.592105 | 439 | 0.085405 | 44.70169 | 124.784897 |
| XPO1 | 7583.510731 | 0.57377 | 638 | 0.06225 | 35.39344 | 89.18553459 |
| HDAC5 | 4146.608095 | 0.570997 | 327 | 0.066555 | 37.87712 | 141.3292308 |
| NTRK1 | 27718.44275 | 0.66879 | 1178 | 0.114246 | 53.43305 | 86.6420068 |
| TP53 | 16668.40004 | 0.621711 | 679 | 0.094548 | 52.15094 | 111.267356 |
| FN1 | 10307.28105 | 0.61284 | 575 | 0.097874 | 48.29226 | 113.0331588 |
| CREBBP | 1907.402322 | 0.541858 | 326 | 0.034135 | 36.375 | 102.2808642 |
| EED | 3555.759817 | 0.570652 | 377 | 0.070077 | 42.97872 | 119.696 |
| YWHAQ | 3348.997615 | 0.554903 | 345 | 0.051841 | 34.32447 | 121.5131195 |
| HSPA4 | 2466.552203 | 0.545297 | 264 | 0.041755 | 28.79618 | 144.2310606 |
| COPS5 | 6349.351221 | 0.597345 | 542 | 0.094921 | 55.50485 | 115.7166667 |
| HSPA5 | 4759.559997 | 0.569277 | 384 | 0.067578 | 45.11255 | 140.6649215 |
| APP | 7295.623413 | 0.56825 | 743 | 0.054994 | 29.27193 | 74.20917679 |
| TRAF6 | 4876.269811 | 0.55621 | 383 | 0.040093 | 24.94792 | 98.98687664 |
| OBSL1 | 5361.220268 | 0.586592 | 462 | 0.077099 | 39.03214 | 113.2956522 |
| SRC | 2050.028842 | 0.536322 | 298 | 0.025259 | 25.66667 | 104.4121622 |
| YWHAZ | 6408.947822 | 0.582255 | 475 | 0.075402 | 50.37313 | 130.0528541 |
| RNF2 | 7900.415838 | 0.587687 | 494 | 0.074351 | 38.40141 | 106.1239837 |
| SMURF1 | 2898.331612 | 0.550058 | 285 | 0.046651 | 24.37356 | 115.6501767 |
| CUL2 | 2697.6453 | 0.564854 | 315 | 0.069731 | 41.45622 | 134.9777778 |
| RPA1 | 3095.218437 | 0.557193 | 330 | 0.055987 | 36.2 | 125.2713415 |
| ARRB2 | 2805.587356 | 0.553927 | 264 | 0.053602 | 30.2973 | 136.1969697 |
| HSPB1 | 1777.525149 | 0.537849 | 302 | 0.038087 | 28.60741 | 118.65 |
| SIRT7 | 4992.296836 | 0.573422 | 468 | 0.067198 | 35.20661 | 103.3012821 |
| RPA3 | 1606.227741 | 0.544983 | 273 | 0.045477 | 29.51592 | 118.3763838 |
| HSPA8 | 4058.046725 | 0.564516 | 350 | 0.060497 | 41.69124 | 145.9252874 |
| TARDBP | 1467.115624 | 0.549099 | 241 | 0.062048 | 50.6954 | 156.251046 |
| CUL1 | 7007.412823 | 0.598101 | 493 | 0.094726 | 56.62058 | 124.4378819 |
| HIST1H3F | 1762.46779 | 0.559834 | 319 | 0.058433 | 48.14634 | 126.1104101 |
| CUL3 | 16038.83547 | 0.641548 | 760 | 0.114238 | 57.70813 | 106.1240106 |
| RPA2 | 2358.850057 | 0.551664 | 307 | 0.05092 | 30.56497 | 120.7947883 |
| HSP90AA1 | 7077.481027 | 0.579399 | 813 | 0.06749 | 44.45769 | 79.22688039 |
| HSP90AB1 | 6001.346925 | 0.572034 | 635 | 0.064576 | 41.25523 | 89.28909953 |
| BRCA1 | 5252.087701 | 0.562835 | 359 | 0.048569 | 31.82629 | 118.6694678 |
| EEF1A1 | 3770.294646 | 0.565191 | 344 | 0.071474 | 47.80822 | 143.9269006 |
| GRB2 | 4725.331891 | 0.559172 | 460 | 0.050938 | 36.16418 | 105.3209607 |
| STAU1 | 2201.115931 | 0.553603 | 252 | 0.057348 | 37.25 | 148.952 |
| RELA | 1931.669304 | 0.537543 | 254 | 0.030901 | 26.46715 | 118.781746 |
| EP300 | 5130.364503 | 0.565868 | 412 | 0.051355 | 40.8371 | 112.6073171 |
| FBXO6 | 3280.603771 | 0.561831 | 368 | 0.057913 | 28.48558 | 112.4782609 |
| BMI1 | 1474.316042 | 0.536932 | 244 | 0.033386 | 23.70149 | 111.7396694 |
| MYC | 4952.550989 | 0.563506 | 454 | 0.048165 | 29.22897 | 99.00221239 |
| CDK1 | 1272.720323 | 0.533898 | 240 | 0.033243 | 26.29752 | 130.2521008 |
| ESR1 | 12580.29497 | 0.605381 | 635 | 0.08769 | 47.80303 | 109.0821485 |
| HIST1H3A | 1762.46779 | 0.559834 | 319 | 0.058433 | 48.14634 | 126.1104101 |
| CDC5L | 4280.046999 | 0.566887 | 401 | 0.06043 | 40.87946 | 116.2606516 |
| COMMD3-BMI1 | 1474.316042 | 0.536932 | 244 | 0.033386 | 23.70149 | 111.7396694 |
| HUWE1 | 5796.631674 | 0.575868 | 356 | 0.067704 | 40.04 | 136.6214689 |
| CUL5 | 1883.148043 | 0.556537 | 289 | 0.065291 | 43.65104 | 137.8650519 |
| CTNNB1 | 3874.433817 | 0.549099 | 291 | 0.038825 | 25.31361 | 130.7525773 |
| SMAD2 | 1553.496908 | 0.531496 | 238 | 0.025538 | 20.47826 | 104.661017 |
| HIST1H3H | 1762.46779 | 0.559834 | 319 | 0.058433 | 48.14634 | 126.1104101 |
| CDK2 | 9999.896478 | 0.602294 | 562 | 0.089226 | 45.47205 | 113.4142857 |
| UBC | 10227.92247 | 0.589888 | 582 | 0.070774 | 43.44983 | 108.9275862 |
| HIST1H3J | 1762.46779 | 0.559834 | 319 | 0.058433 | 48.14634 | 126.1104101 |
| CAND1 | 5156.489161 | 0.588418 | 470 | 0.091431 | 58.93357 | 120.3974359 |
| IKBKG | 1719.425348 | 0.539692 | 334 | 0.035861 | 27.38571 | 100.4789157 |
| CUL7 | 8177.696597 | 0.603448 | 516 | 0.089112 | 43.54462 | 116.3735409 |
| HIST1H3I | 1762.46779 | 0.559834 | 319 | 0.058433 | 48.14634 | 126.1104101 |
| HIST1H3D | 1762.46779 | 0.559834 | 319 | 0.058433 | 48.14634 | 126.1104101 |
| UBE2I | 3410.408494 | 0.549738 | 344 | 0.040323 | 27.60465 | 107.002924 |
| HIST1H3E | 1762.46779 | 0.559834 | 319 | 0.058433 | 48.14634 | 126.1104101 |
| SNW1 | 5069.825262 | 0.564854 | 370 | 0.054819 | 37.02294 | 120.9266304 |
| HIST1H3B | 1762.46779 | 0.559834 | 319 | 0.058433 | 48.14634 | 126.1104101 |
| SMAD3 | 1733.049032 | 0.535411 | 250 | 0.027331 | 25.03937 | 113.7741936 |
| SUZ12 | 2756.551615 | 0.551664 | 278 | 0.045472 | 30.70879 | 120.2137681 |
| EWSR1 | 3949.730045 | 0.55621 | 361 | 0.052183 | 32.05729 | 118.724234 |
| HDAC1 | 2753.554644 | 0.551986 | 367 | 0.042441 | 38.51381 | 107.1945206 |
| HIST1H3G | 1762.46779 | 0.559834 | 319 | 0.058433 | 48.14634 | 126.1104101 |
| HIST1H3C | 1762.46779 | 0.559834 | 319 | 0.058433 | 48.14634 | 126.1104101 |
| UBL4A | 1872.447079 | 0.546875 | 237 | 0.055752 | 41.83133 | 133.7890295 |
| VCAM1 | 5054.221408 | 0.584054 | 395 | 0.079842 | 39.8315 | 125.3587786 |
| PARK2 | 3791.810598 | 0.560166 | 331 | 0.053325 | 30.07353 | 120.6443769 |
| U2AF2 | 3062.439067 | 0.559834 | 294 | 0.058445 | 43.4532 | 143.239726 |
| FUS | 2750.862601 | 0.558511 | 267 | 0.06279 | 43.95477 | 165.4792453 |
| NPM1 | 7278.017279 | 0.595463 | 456 | 0.098278 | 66.08882 | 146.6211454 |
| MDM2 | 4308.120149 | 0.55621 | 332 | 0.048206 | 30.45313 | 121.6515152 |
| CCDC8 | 3999.177241 | 0.569964 | 392 | 0.065569 | 33.5279 | 116.6641026 |
| HNRNPA1 | 3044.084427 | 0.565868 | 346 | 0.071855 | 54.91403 | 146.5930233 |
| AKT1 | 1914.190705 | 0.537543 | 270 | 0.028878 | 24.07519 | 114.0485075 |
| VCP | 4916.072183 | 0.566547 | 411 | 0.061855 | 40.95516 | 128.195599 |
| PAN2 | 2985.903039 | 0.559503 | 281 | 0.063711 | 35.3399 | 127.8185053 |
| MCM2 | 11993.58698 | 0.619266 | 609 | 0.103951 | 56.1863 | 119.446458 |
| HNRNPU | 5163.085903 | 0.580467 | 379 | 0.086641 | 63.03053 | 142.8970976 |
| VHL | 3728.448111 | 0.557522 | 329 | 0.054708 | 29.89286 | 119.7492355 |
| RPS27A | 2655.789762 | 0.547826 | 245 | 0.052402 | 37.875 | 151.2427984 |
